# Sema7A, a brain immune regulator, regulates seizure activity in PTZ‐kindled epileptic rats

**DOI:** 10.1111/cns.13181

**Published:** 2019-06-09

**Authors:** Jing Deng, Tao Xu, Juan Yang, Ke‐Ming Zhang, Qi Li, Xin‐Yuan Yu, Rong Li, Jie Fu, Qian Jiang, Jing‐Xi Ma, Yang‐Mei Chen

**Affiliations:** ^1^ Department of Neurology The Second Affiliated Hospital of Chongqing Medical University Chonqing China; ^2^ Department of Neurology, Chongqing General Hospital University of Chinese Academy of Sciences Chonqing China; ^3^ Chongqing Key Laboratory of Neurodegenerative Diseases Chongqing China

**Keywords:** epilepsy, IL‐6, mossy fibers, neuroinflammation, semaphorin7A, TNF‐α

## Abstract

**Aims:**

Semaphorin7A (Sema7A) plays an important role in the immunoregulation of the brain. In our study, we aimed to investigate the expression patterns of Sema7A in epilepsy and further explore the roles of Sema7A in the regulation of seizure activity and the inflammatory response in PTZ‐kindled epileptic rats.

**Methods:**

First, we measured the Sema7A expression levels in patients with temporal lobe epilepsy (TLE) and in rats of a PTZ‐kindled epilepsy rat model. Second, to explore the role of Sema7A in the regulation of seizure activity, we conducted epilepsy‐related behavioral experiments after knockdown and overexpression of Sema7A in the rat hippocampal dentate gyrus (DG). Possible Sema7A‐related brain immune regulators (eg, ERK phosphorylation, IL‐6, and TNF‐α) were also investigated. Additionally, the growth of mossy fibers was visualized by anterograde tracing using injections of biotinylated dextran amine (BDA) into the DG region.

**Results:**

Sema7A expression was markedly upregulated in the brain tissues of TLE patients and rats of the epileptic model after PTZ kindling. After knockdown of Sema7A, seizure activity was suppressed based on the latency to the first epileptic seizure, number of seizures, and duration of seizures. Conversely, overexpression of Sema7A promoted seizures. Overexpression of Sema7A increased the expression levels of the inflammatory cytokines, IL‐6 and TNF‐α, ERK phosphorylation, and growth of mossy fibers in PTZ‐kindled epileptic rats.

**Conclusion:**

Sema7A is upregulated in the epileptic brain and plays a potential role in the regulation of seizure activity in PTZ‐kindled epileptic rats, which may be related to neuroinflammation. Sema7A promotes the inflammatory cytokines TNF‐α and IL‐6 as well as the growth of mossy fibers through the ERK pathway, suggesting that Sema7A may promote seizures by increasing neuroinflammation and activating pathological neural circuits. Sema7A plays a critical role in epilepsy and could be a potential therapeutic target for this neurological disorder.

## 1 BACKGROUND

Epilepsy, currently one of the most common chronic neurological disorders, is characterized by persistent brain susceptibility to generating epileptic seizures. Up to ~1% of people worldwide suffer from epilepsy,^1^ and temporal lobe epilepsy (TLE), the most common type of focal epilepsy, has the highest incidence rate compared with other seizure types as well as a high refractory rate, leading to an elevated risk of neurological deficits.^2^ TLE features a variety of molecular and anatomical changes, including inflammation, mossy fiber sprouting, neuronal death, neurogenesis, gliosis, and neuronal network remodeling.^3^, ^4^, ^5^

Recently, numerous studies have suggested that the immune system and inflammatory processes in the central nervous system (CNS) may be important mechanisms involved in the pathophysiology of epilepsy and seizures.^6^, ^7^, ^8^ Downstream inflammatory cytokines such as interleukin IL‐1β, IL‐6, tumor necrosis factor (TNF)‐α, and prostaglandin E2 (PGE2), as well as some inflammatory mediators including nuclear factor kappa B (NF‐κB) and cyclooxygenase (COX)‐2, are activated in the progression of seizures.^9^ In the PTZ‐kindled epileptic rat model, increased expression levels of IL‐6 and TNF‐α were found.^10^, ^11^, ^12^ Epileptic seizures induce the release of IL‐6 and TNF‐α cytokines from glial cells, likely increasing neuronal cell loss, enhancing extraneuronal glutamate concentrations, and decreasing K^+^ and glutamate uptake by glia. Such inflammatory responses cause hyperexcitability of neurons and recurrent seizures, eventually leading to the development of refractory epilepsy.^13^, ^14^, ^15^, ^16^ Furthermore, the mitogen‐activated protein kinase (MAPK) signaling pathway is known to play an important role in epilepsy. The phosphorylation of extracellular regulated protein kinase (ERK) was significantly increased in the brains of TLE patients compared with that in the brains of controls, suggesting that ERK is activated in epilepsy.^17^ In PTZ‐kindled rats, inhibiting the TGF‐β‐mediated ERK signaling pathway can alleviate inflammation responses in epilepsy.^18^ However, the mechanism by which inflammation is involved in the pathogenesis of epilepsy remains unclear and requires further exploration.

Semaphorins (Semas), consisting of 20 family members in vertebrates, are one of the largest families of guiding clues and have a variety of important functions in the peripheral and CNSs. Five types of semaphores exist, named classes 3, 4, 5, 6, and 7.^19^, ^20^ Semaphorin7A (Sema7A) is the only membrane‐associated glycosylphosphatidylinositol (GPI)‐linked semaphorin and plays a role in the regulation of neuronal axon outgrowth. Importantly, Sema7A also mediates the immune response in multiple systems, such as the lungs, liver, eyes, and bones.^21^, ^22^, ^23^, ^24^ Emerging evidence suggests that Sema7A not only promotes inflammatory responses by inducing proinflammatory cytokines, such as TNF‑α and IL‑6, but also acts as a potent autocrine stimulator of monocytes, stimulating chemotaxis and inflammatory cytokine production and superoxide release.^25^ Sema7A initiates inflammatory responses by inducing phosphorylation of ERK 1 and ERK 2, as indicated by research results obtained using a human monocytic cell line.^26^ In the CNS, Sema7A is expressed in multiple regions, including the hippocampus, olfactory system, hypothalamic‐pituitary system, mesodiencephalic dopamine system, and spinal cord.^27^ Sema7A is reportedly involved in CNS inflammation in experimental autoimmune encephalomyelitis (EAE) and multiple sclerosis (MS).^28^ In neuronal cells, Sema7A also activates MAPK signaling cascades, which are derived from the β1 integrin receptor and affect processes of neuronal network formation, such as axonal and dendritic growth, branching, guidance and pruning, target recognition, and synapse formation.^29^, ^30^ These characteristics of Sema7A suggest that it functions in a variety of ways to regulate neuroinflammation and axon guidance, which may lead to epileptogenesis.

The role of Sema7A in epilepsy remains unknown. In this study, the effects of Sema7A on TLE patients and epileptic rat models were initially investigated. First, we investigated the protein expression of Sema7A in PTZ‐kindled epileptic rats and in TLE patients. Additionally, we analyzed behavioral activity and hippocampal morphological changes in PTZ‐kindled rats after lentivirus‐mediated knockdown and overexpression of Sema7A. Furthermore, the mechanism underlying Sema7A‐related inflammation in epilepsy was explored.

## 2 METHOD

### 2.1 Human brain tissues

Sixteen brain tissue samples from refractory TLE patients were obtained from the Second Affiliated Hospital of Army Medical University (Chongqing Third Military Medical University).^31^ All human‐related experiments were performed in accordance with the World Medical Association's Code of Ethics (Helsinki Declaration) and the ethical principles and guidelines of the National Institutes of Health, and were approved by the Ethics Committee of Chongqing Medical University, China. Prior to the surgery, all patients or their guardians were informed of the use of brain tissue in the research and then provided informed consent. According to diagnostic criteria proposed by the International League Against Epilepsy (ILAE),^32^ the patients with refractory TLE had typical clinical symptoms and electroencephalogram (EEG) performance, and their epileptic seizures could not be successfully controlled despite the combined use of three or more types of antiepileptic drugs (AEDs) at the maximum dose. Preoperative assessment included detailed medical history and neurological examination, neuroradiology studies, ictal and interictal EEG recordings, and neuropsychological testing. The epileptogenic zone in the temporal neocortex was removed from the brain of each TLE patient. The clinical features of the TLE patients are listed in Table 1.

**Table 1:** Clinical features of TLE patients

No.	Sex	Age at surgery (y)	Course (y)	AEDs before surgery	Resected tissue	Pathologic result
1	F	5	2	OXC, CLZ, VPA, TPM	LTN	G
2	F	14	11	OXC, VPA, PHT	RTN	G, NL, ND
3	M	39	7	CBZ, PHT, VPA	RTN	G, NL, ND
4	M	27	4	CBZ, VPA, CLZ	LTN	G, NL
5	M	22	8	CBZ, VPA, PB	LTN	G, NL, ND
6	F	9	4	OXC, VPA, GBP	RTN	G, NL
7	M	27	4	CBZ, VPA, PHT	LTN	G, NL
8	M	26	14	CBZ, VPA, TPM	RTN	G, NL, ND
9	F	22	11	OXC, VPA, TPM	RTN	G, NL, ND
10	M	13	7	OXC, PHT, VPA	RTN	G, NL, ND
11	F	35	17	CBZ, VPA, CLZ, TPM	LTN	G, NL, ND
12	M	17	9	OXC, VPA, PHT	LTN	G, NL, ND
13	M	23	6	CBZ, PHT, TPM	RTN	G, NL
14	F	4	2	OXC, PHT, TPM	LTN	G
15	F	18	6	OXC, VPA, LTG	LTN	G, NL
16	M	31	8	CBZ, PHT, LTG	RTN	G, NL, ND

Abbreviations: AEDs, antiepileptic drugs; CBZ, carbamazepine; CLZ, clonazepam; F, female; G, gliosis; M, male; ND, neuronal degeneration; NL, neuronal loss; LTG, lamotrigine; LTN, left temporal neocortex; PB, phenobarbitone; PHT, phenytoinGBP, gabapentin; RTN, right temporal neocortex; TPM, topiramate; OXC, oxcarbazepine; VPA, valproic acid.

As the control group, twelve brain tissue samples were also obtained from the Second Affiliated Hospital of Army Medical University. We obtained temporal neocortex samples from patients who underwent craniotomy due to severe traumatic brain injury. Patients of the control group had no history of epilepsy or any other neurological disease and had never used AEDs. Pathological diagnosis revealed that all resected brain tissues were normal. The clinical features of the control patients are listed in Table 2.

**Table 2:** Clinical features of control patients

No.	Sex	Age	Disease diagnosis	Resected tissue	Pathologic result
1	M	28	Brain trauma	LTN	Normal
2	F	22	Brain trauma	LTN	Normal
3	F	13	Brain trauma	RTN	Normal
4	M	17	Brain trauma	RTN	Normal
5	M	23	Brain trauma	LTN	Normal
6	F	28	Brain trauma	RTN	Normal
7	F	18	Brain trauma	LTN	Normal
8	M	33	Brain trauma	RTN	Normal
9	F	15	Brain trauma	LTN	Normal
10	F	31	Brain trauma	RTN	Normal
11	M	44	Brain trauma	LTN	Normal
12	M	16	Brain trauma	LTN	Normal

Abbreviations: F, female; LTN, left temporal neocortex; M, male; RTN, right temporal neocortex.

### 2.2 Experimental animals

Adult male Sprague‐Dawley rats (200‐230 g) were obtained from the Experimental Animal Center of Chongqing Medical University. The animals were housed in a specific‐pathogen‐free (SPF) animal facility at constant temperature (22‐24°C) and humidity (55‐65%) under an artificial 12/12‐hours dark‐light cycle. Food and water were available ad libitum. All animal experimental procedures and applicable regulations on animal welfare were in accordance with the guidelines of the Animal Welfare Agency of Chongqing Medical University and were approved by the Ethical Committee for Experimental Animals.

### 2.3 Pentylenetetrazole (PTZ) kindling

The rats received a daily subconvulsive dose of PTZ (35 mg/kg, Sigma‐Aldrich), and their behaviors were then observed for 30 minutes in a Plexiglas chamber. Seizure behavior was monitored according to Racine's standard criteria as follows: grade 0, no response; grade 1, facial myoclonus; grade 2, head nodding; grade 3, forelimb clonus; grade 4, rearing and severe forelimb clonus; grade 5, rearing, falling, and severe forelimb clonus; and grade 6, death.^33^ Only rats with at least three consecutive seizures of grade 4 or 5 within 28 days were considered fully kindled and were assigned to the epilepsy group. The controls, in contrast, received an equal amount of saline instead of PTZ. The PTZ injections were administered between 9:00 am and 12:00 am to minimize possible complicating effects on the circadian rhythms of the rats.

### 2.4 Recording of local field potentials (LFPs) during PTZ‐induced chronic seizures

Each rat was deeply anesthetized by intraperitoneal injection of pentobarbital (60 mg/kg) and placed on a stereotaxic apparatus (RWD Life Science, Shenzhen, China). Then, a U‐shaped frame was fixed to the skull to hold the head.^34^ Before LFP recording, electrodes were implanted into the dorsal hippocampus (anterior‐posterior: −3.6 mm; medial‐lateral: −2.8 mm; dorsal‐ventral: −3.0 mm) and attached to an LFP signal connector, which was fixed to the skull using dental acrylic cement. LFPs were recorded with a MAP data acquisition system (Plexon) after the last PTZ injection. The signals were filtered (0.1‐500 Hz), preamplified (1000×), and digitized at 4 kHz. The recordings were carried out after the rats had been successfully kindled according to Racine's classification.

### 2.5 Lentiviral vector production

Lentiviral vectors expressing Sema7A (Sema7A) and containing an enhanced green fluorescent protein (EGFP) sequence were produced by GeneCopoeia Inc. The same lentiviral vectors, encoding only EGFP, were used as controls (Con‐Sema7A). For Sema7A knockdown, we constructed lentiviral vectors expressing Sema7A RNAi (Sema7A‐RNAi), which was designed and synthesized based on the following sequence: GCCATAACCGCACTGTCATTC. The same lentiviral vectors, but coding for EGFP (Con‐RNAi), were used as the control. The viruses were prepared according to a previously described method.^35^ The titer of the lentiviral vectors for transfection was 2 × 10^8^ TU/mL.

### 2.6 Stereotaxic injections and U0126 treatment

Stereotaxic injections were performed as described previously with minor modifications.^36^ Rats were deeply anesthetized by intraperitoneal injection of pentobarbital (60 mg/kg) and placed in a stereotaxic frame (RWD Life Science). A volume of 2.5 μL of Sema7A, Con‐Sema7A, Sema7A‐RNAi, or Con‐RNAi was infused through a glass pipette (0.2 μL/min) bilaterally into the hippocampus region (anterior‐posterior: −3.3 mm; medial‐lateral: ±2.0 mm; dorsal‐ventral: −3.5 mm). To prevent backflow, the pipette was retained in the hippocampus for at least 5 minutes and then retracted steadily and slowly. To ensure effective expression of the lentivirus, Western blot analysis was conducted at 2 weeks after injection into the transfected brain tissues of rats. PTZ was administered for kindling starting at 14 days after lentivirus injection.

U0126 (MedChemExpress) is a specific inhibitor of MAPK kinase (MEK‐1 and MEK‐2) that effectively blocks ERK1/2 phosphorylation. U0126 was dissolved in 1% dimethyl sulfoxide (DMSO) solvent, and 30 mg/kg was injected intraperitoneally once per day.^37^, ^38^ As described in Figure 9A, the intraperitoneal injections were started 3 days before the initial administration of PTZ, and U0126 was administered 1 hour before each PTZ injection, while the same volume of DMSO was administered to the vehicle group.

### 2.7 Behavioral assessment

Two weeks after lentivirus injection, each group was given a daily dose of PTZ (35 mg/kg, ip,), and behavioral changes were accessed. Rats with grade 4 or 5 seizures were selected for behavioral studies, and the latency to the first seizure, number of seizures, and total duration of seizures were separately recorded.^39^, ^40^

Two weeks after lentivirus (Sema7A) injection, U0126 or DMSO was intraperitoneally injected once per day. PTZ kindling was performed at 3 days after U0126 injection. Behavioral assessments were performed in Sema7A + U0126+PTZ and Sema7A + DMSO + PTZ groups. (n = 10 biological replicates).

### 2.8 Tissue preparation

#### 2.8.1 Human

One portion of each brain tissue sample was immediately stored in liquid nitrogen for Western blot analysis. Another portion of each sample was immersed in 4% paraformaldehyde for 48 hours. Then, a portion of the fixed tissue was removed for paraffin embedding and sliced at a thickness of 5 μm for immunohistochemistry analysis. The remaining tissues were frozen and sliced at 10 μm for immunofluorescence analysis. (n = 5 biological replicates).

#### 2.8.2 Rat

The rats were anesthetized with pentobarbital (60 mg/kg), and the brains of one subset of rats were removed. The hippocampus and cortex were collected and stored in liquid nitrogen for Western blot analysis. The other rats were intracardially perfused with 0.9% saline and 4% paraformaldehyde, and their brains were removed and fixed in paraformaldehyde for 24 hours. Some fixed brains were frozen and sliced at a thickness of 15 μm for immunofluorescence analysis, and the other samples were embedded in paraffin and sliced at a thickness of 5 μm for immunohistochemistry analysis. To detect GFP fluorescence as a sign of successful lentivirus transfection, the brain tissues transfected with lentivirus were sliced into 20‐μm‐thick sections at −20°C for immunofluorescence analysis and mounted on polylysine‐coated slides for observation on a laser confocal microscope. To determine the expression of Sema7A in the lentivirus‐transfected rats, brain samples from the Sema7A, Con‐Sema7A, Sema7A‐RNAi, and Con‐RNAi groups were acquired at 14 and 42 days after injection and stored in liquid nitrogen for Western blotting. (n = 5 biological replicates).

### 2.9 Western blotting

Total protein from brain tissues was extracted using a total protein extraction kit (Beyotime), and the protein concentration was detected using a bicinchoninic acid (BCA) Protein Concentration Assay Kit (Beyotime) according to the manufacturer's instructions. The samples were denatured and stored at −8°C. Protein was separated by SDS‐PAGE with a 5% stacking gel and a 10% separating gel and then transferred to a PVDF membrane (Millipore). After being blocked with 5% defatted milk for 1 hour at 37°C, the membranes were incubated at 4°C overnight with the following primary antibodies: anti‐Sema7A rabbit polyclonal antibody (1:1000, GeneTex), anti‐pERK1/2 rabbit monoclonal antibody (1:2000, Cell Signaling Technology), anti‐ERK1/2 rabbit monoclonal antibody (1:2000, Cell Signaling Technology), anti‐TNFα mouse monoclonal antibody (1:2000, Proteintech), and anti‐IL6 rabbit polyclonal antibody (1:1000, Proteintech). The anti‐GAPDH rabbit polyclonal antibody (1:3000, Proteintech) and anti‐α‐tubulin rabbit polyclonal antibody (1:2000, Proteintech) were used as internal controls. Then, the membranes were washed with 1 × TBST for 5 minutes three times and incubated with a horseradish peroxidase‐conjugated goat anti‐rabbit IgG antibody (1:4000, Proteintech) or horseradish peroxidase‐conjugated goat anti‐mouse IgG antibody (1:4000, Proteintech) for 1 hour at room temperature. After washing, the immunoreactivity on the membrane was visualized by enhanced chemiluminescence (ECL) substrates. Quantity One software (Bio‐Rad) was used to analyze the data.^38^ The experiments were repeated three times under the same conditions. The final intensity ratio was obtained by averaging the three experimental values.

### 2.10 Immunofluorescence labeling

Frozen sections were dried at room temperature and then permeabilized with 0.4% Triton X‐100. The antigen was retrieved by heating in a microwave oven for 20 minutes at 98°C. After being washed with phosphate‐buffered saline (PBS) three times, the sections were blocked in 5% goat serum (Boster Biological Technology) for 1 hour at room temperature and then incubated with a mixture of a rabbit polyclonal anti‐Sema7A antibody (1:50, GeneTex) and an anti‐neuron‐specific enolase (NSE) mouse monoclonal antibody (1:50, Proteintech) or with a mixture of a rabbit polyclonal anti‐Sema7A antibody (1:50) and mouse monoclonal anti‐glial fibrillary acidic protein (GFAP) antibody (1:100, Boster Biological Technology) at 4°C overnight. The sections were washed with PBS and incubated with a mixture of fluorescein isothiocyanate (FITC)‐conjugated goat anti‐rabbit IgG (1:100, Beyotime) and Cy3‐labeled goat anti‐mouse IgG (1:200, Beyotime, Shanghai, China) in the dark for 60 minutes at 37°C. Then, the sections were washed and counterstained with 10% 4',6‐diamidino‐2‐phenylindole (DAPI). After being mounted in 50% glycerol/PBS, the sections were visualized by laser scanning confocal microscopy (Leica Microsystems Heidelberg GmbH). The quantitative analysis of Sema7A expression was carried out using Image‐Pro Plus 6.0 software (Media Cybernetics), while the mean density value was obtained by calculating the ratio of the integrated optical density (IOD) and the selected corresponding area.^41^

### 2.11 Immunohistochemistry

All experiments were performed under the same conditions and repeated three times. Sections were deparaffinized in xylene and rehydrated in graded ethanol, and antigen was then retrieved by microwave for 20 minutes at 98°C with 10 mmol/L sodium citrate buffer. After incubation with 0.3% H_2_O_2_ at 37°C for 15 minutes, the sections were incubated with a primary rabbit polyclonal anti‐Sema7A antibody (1:50, GeneTex) at 4°C overnight. After washing with PBS, the sections were incubated with the corresponding secondary antibodies (horseradish peroxidase‐conjugated monoclonal goat anti‐rabbit IgG; PV9001, Zhongshan Golden Bridge Inc) for 1 hour at room temperature. Immunohistochemistry (EnVision) analysis was performed according to the manufacturer's protocol. After washing with PBS, the sections were incubated with 3,3′‐diaminobenzidine (DAB, Zhongshan Golden Bridge Inc) to develop the positive signals, and distilled water was used to stop the reaction. The sections were counterstained with hematoxylin, dehydrated and transparently sealed. Cells with buffy staining in the membrane and cytoplasm were identified as Sema7A‐positive cells. Five visual fields were randomly selected for each sample under the microscope (Leica DM600B Microsystems Heidelberg GmbH). Sema7A‐positive cells were quantified using Image‐Pro Plus 6.0 (Media Cybernetics), and the mean density value for each visual field was calculated from the ratio of the IOD value and the selected corresponding area.

### 2.12 Biotinylated dextran amine (BDA) tracing

After PTZ kindling, rats were deeply anesthetized with pentobarbital (60 mg/kg), and 5 μL of BDA (10 000 MW; Invitrogen) was stereotaxically injected into the dentate gyrus (DG) region of the hippocampus (coordinates from Bregma: anterior‐posterior: −3.6 mm; medial‐lateral: −2.5 mm; dorsal‐ventral: −3.5 mm) using a microsyringe.

Fourteen days after BDA injection, rats were anesthetized with pentobarbital and perfused transcardially with 4% paraformaldehyde in PBS. The brains were removed, fixed overnight in 4% paraformaldehyde, dehydrated in sucrose, frozen, and sectioned at 40 μm. The sections were processed to reveal BDA‐positive fibers by incubation with an avidin‐biotin complex (ABC; BDA‐10,000 Neuronal Tracer Kit, Invitrogen) solution (1.0 µg/mL) in PBS containing 0.3% Triton X‐100 at 4°C overnight. The sections were treated with 0.03% DAB and 0.004% hydrogen peroxide. The procedure was performed as previously described.^42^ Mossy fiber pathways, indicated by BDA‐positive fibers, were visualized under a microscope (Leica DM600B Microsystems Heidelberg GmbH) with different objectives.

### 2.13 Statistical analysis

All values are normally distributed according to the Kolmogorov‐Smirnov test. Homogeneity of variance was carried out with Levene's test. Student's *t* test and the chi‐squared test were used for comparisons of age and sex in TLE patients and controls. The other statistical results were analyzed by a two‐tailed unpaired Student's *t* test or one‐way analysis of variance (ANOVA) followed by Dunnett's test. All data are presented as the mean ± standard deviation (SD). The software programs SPSS 20.0 (IBM) and GraphPad Prism 7 (GraphPad software) were used for statistical analyses and graphing. The significance was set at *P* < 0.05.

## 3 RESULTS

### 3.1 Clinical characteristics of TLE and control subjects

The average age of the sixteen TLE patients was 20.75 ± 2.541 (nine males and seven females), and the average age of the twelve controls was 24 ± 2.637 (six males and six females). Analysis of gender distribution (chi‐squared = 0.1077, *P* = 0.7428) and age distribution (*P* = 0.3903) revealed no significance in either the TLE group or the control group.

### 3.2 Sema7A expression in human brain tissues

**Figure 1:**
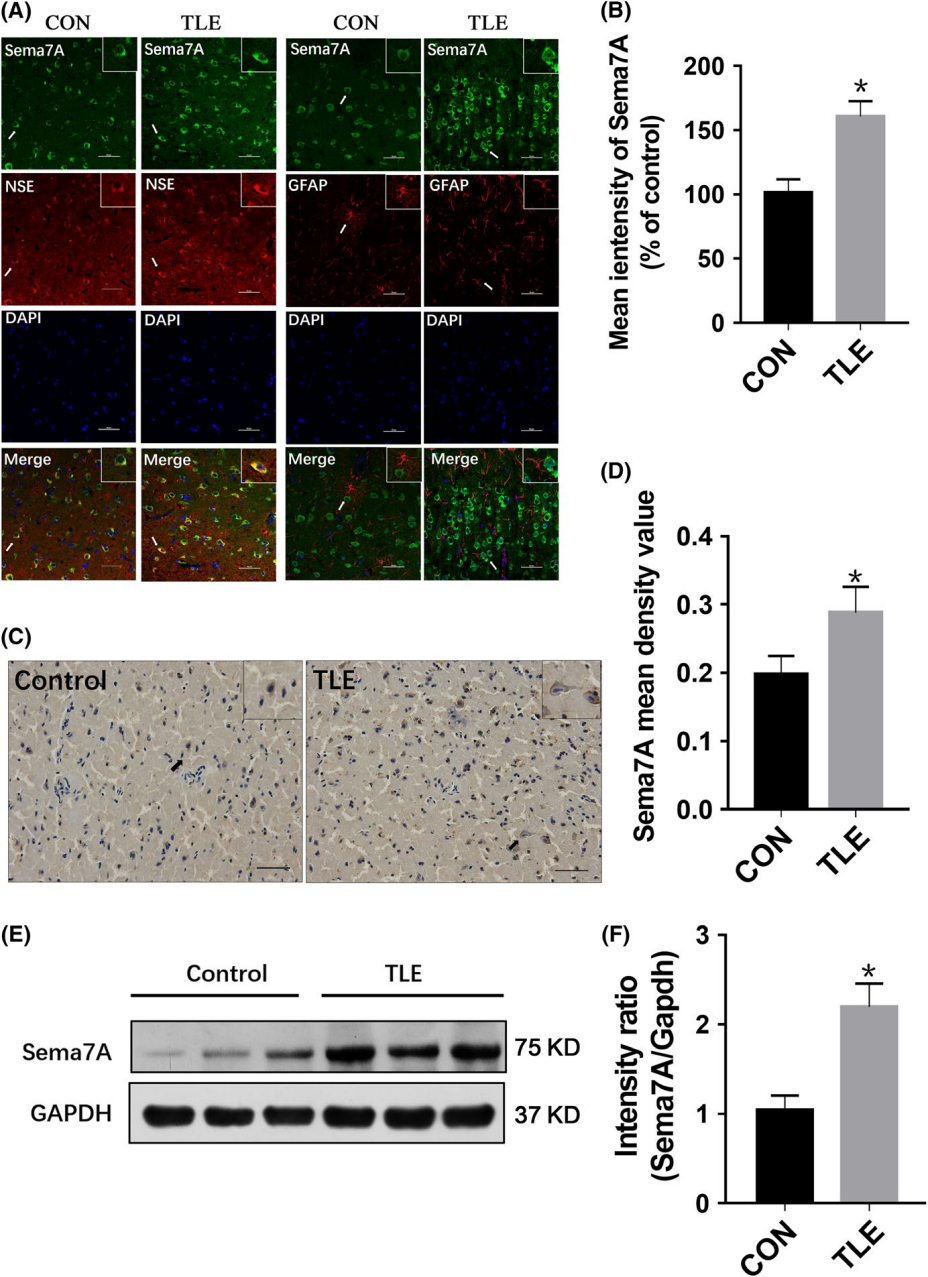
Sema7A expression in TLE patients and nonepileptic patients. A, Images of double immunofluorescence for Sema7A (green). In the human temporal neocortex, Sema7A and NSE (red) are coexpressed (merged), and Sema7A and GFAP (red) are not coexpressed (merged). DAPI staining (blue) represents the cellular nucleus. Compared with that in the control group, the fluorescence intensity of Sema7A in the temporal cortex of TLE patients was stronger. Arrows show the positive cells (scale bar = 100 μm). B, The mean intensity value of Sema7A immunofluorescence (% of control) in the temporal neocortex of the TLE group was significantly higher than that in the control group. C, Images of immunohistochemical staining for Sema7A. Stronger immunoreactive staining of Sema7A in the temporal neocortex is shown in TLE patients than in the control group. Arrows show the positive cells (scale bar = 50 μm). D, The mean immunohistochemical density value of Sema7A was significantly higher in TLE patients than in the control group. E, Representative Western blot images of Sema7A expression (75 kDa) in the human temporal neocortex. F, The intensity of Sema7A in the Western blot indicates significantly higher expression in the epilepsy group than in the control group. (*P < 0.05, unpaired *t* test; n = 5)

To determine the cellular localization and distribution of Sema7A in human brain tissue, we used double immunofluorescence labeling and immunohistochemistry. In human brains, Sema7A was mainly costained with a neuron‐specific marker NSE but not with the astrocyte marker GFAP in the TLE and control groups, suggesting that Sema7A was mainly expressed in the cell membrane and cytoplasm of neurons but not in astrocytes in the human temporal neocortex (Figure 1A). Quantitative analysis of immunofluorescence showed a stronger intensity in the TLE group than in the control group (Figure 1B). Immunohistochemical analysis also showed that Sema7A was present in the cell membrane and cytoplasm (Figure 1C), and the staining of Sema7A in the brain tissues of TLE patients was significantly stronger than in brain tissues of the control group (*P* < 0.05; Figure 1D). Western blot analysis was used to further detect the expression of Sema7A in the temporal neocortex of TLE patients and controls (Figure 1E), revealing that the expression of Sema7A was increased significantly in TLE patients compared with that in control group patients (*P* < 0.05; Figure 1F).

### 3.3 Sema7A expression in PTZ‐kindled epileptic rat models

**Figure 2:**
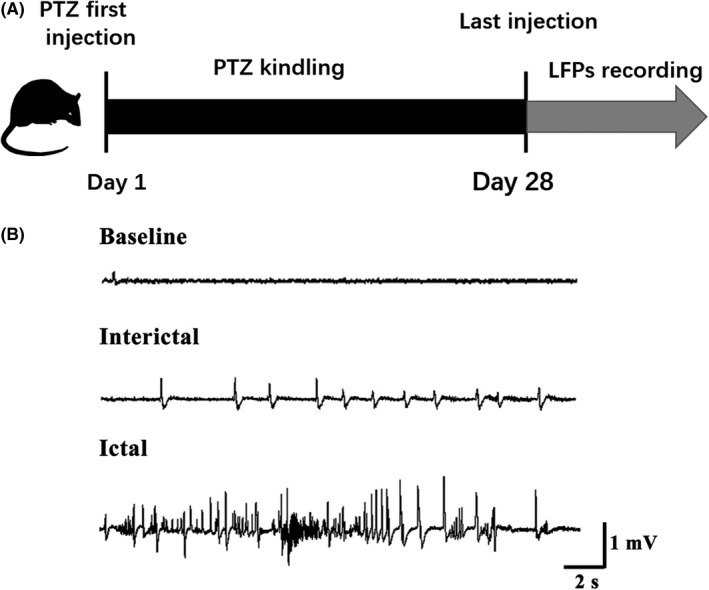
LFP recordings from rats after PTZ kindling. A, Schematic diagram of the experimental design. Electrodes were implanted into the dorsal hippocampus after the last PTZ injection, and the LFP was then recorded. B, Representative LFP recording from a successfully kindled rat. The baseline, interictal, and ictal periods were recorded. Seizure spikes were detected in both the interictal and ictal periods, indicating that the PTZ models were successful

**Figure 3:**
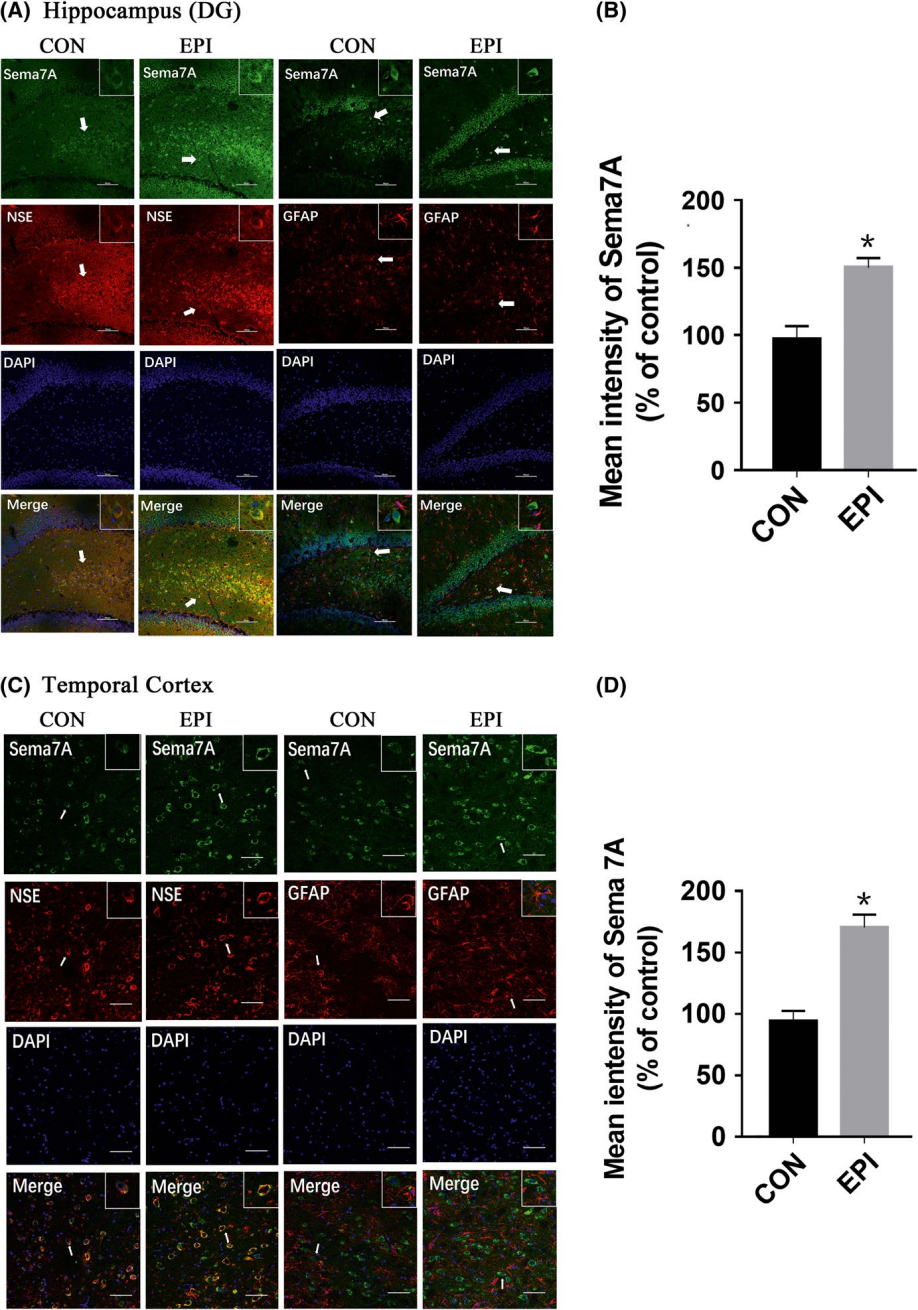
Double immunofluorescence labeling of Sema7A in rat tissues. A, C, In both the hippocampus (A) and adjacent temporal cortex (C) of the epileptic rat model, Sema7A (green) and NSE (red) are coexpressed (merged), while Sema7A and GFAP (red) are not coexpressed (merged). Arrows show the positive cells (scale bar = 100 μm). B, D, In the hippocampus (B) and adjacent temporal cortex (D), the mean intensity values of Sema7A were significantly higher in the epilepsy group (EPI) than in the control group. (*P < 0.05, unpaired *t* test, n = 5)

To further investigate the expression of Sema7A in epilepsy, we detected the expression level of Sema7A in PTZ‐kindled rat models of epilepsy. After the rats were kindled by injection of a subconvulsive dose of PTZ (35 mg/kg) every day for 28 days, LFPs were recorded to assess epileptiform discharges in the PTZ‐induced epileptic rat model (Figure 2A). In this model, we detected epileptiform discharge during an episode of behavioral seizure (Figure 2B). As revealed by double immunofluorescence labeling analysis, Sema7A was observed in the cell membrane and cytoplasm of neurons in the DG region (Figure 3A) and temporal cortex (Figure 3C).

**Figure 4:**
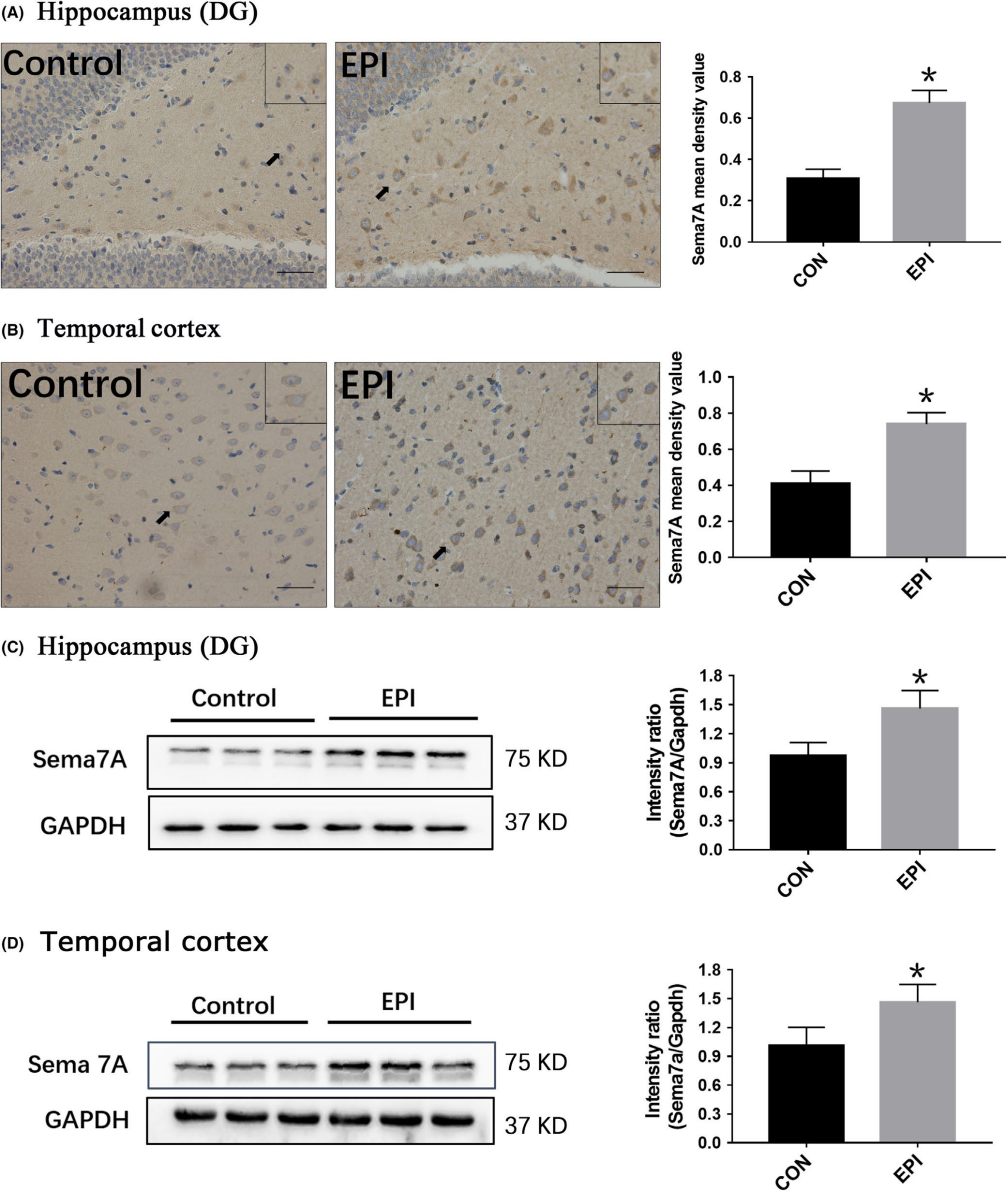
Immunohistochemical and Western blot analysis of Sema7A in rat tissues. A, B Images (left) and statistical graphs (right) of immunohistochemical staining. Compared with that in the control group (control), Sema7A immunoreactive staining was stronger in the hippocampus (A) and adjacent cortex (B) of the epilepsy group (EPI) after PTZ kindling. Arrows show the positive cells (scale bar = 50 μm). C, D, Representative Western blot images (left) and statistical graphs (right) of Sema7A in the hippocampus (C) and adjacent temporal cortex (D) of rats from the EPI and control groups. The mean intensity ratio of Sema7A in the epilepsy group was significantly higher than that in the control group. (*P < 0.05, unpaired *t* test, n = 5)

Quantitative analysis of immunofluorescence showed that the mean intensity of Sema7A in the hippocampus (Figure 3B) and adjacent temporal cortex (Figure 3D) of epileptic rats was increased, indicating that the expression of Sema7A in the hippocampus of epileptic rats was significantly higher than that in control group rats (*P* < 0.05). Furthermore, the location of Sema7A between the two groups revealed no difference. Similar results were also obtained by immunohistochemistry analysis, as Sema7A was observed in the cell membrane and cytoplasm, and enhanced staining of Sema7A was detected in the DG region of the hippocampus (Figure 4A) and temporal cortex.

### 3.4 Sema7A expression after transfection with recombinant lentivirus

**Figure 5:**
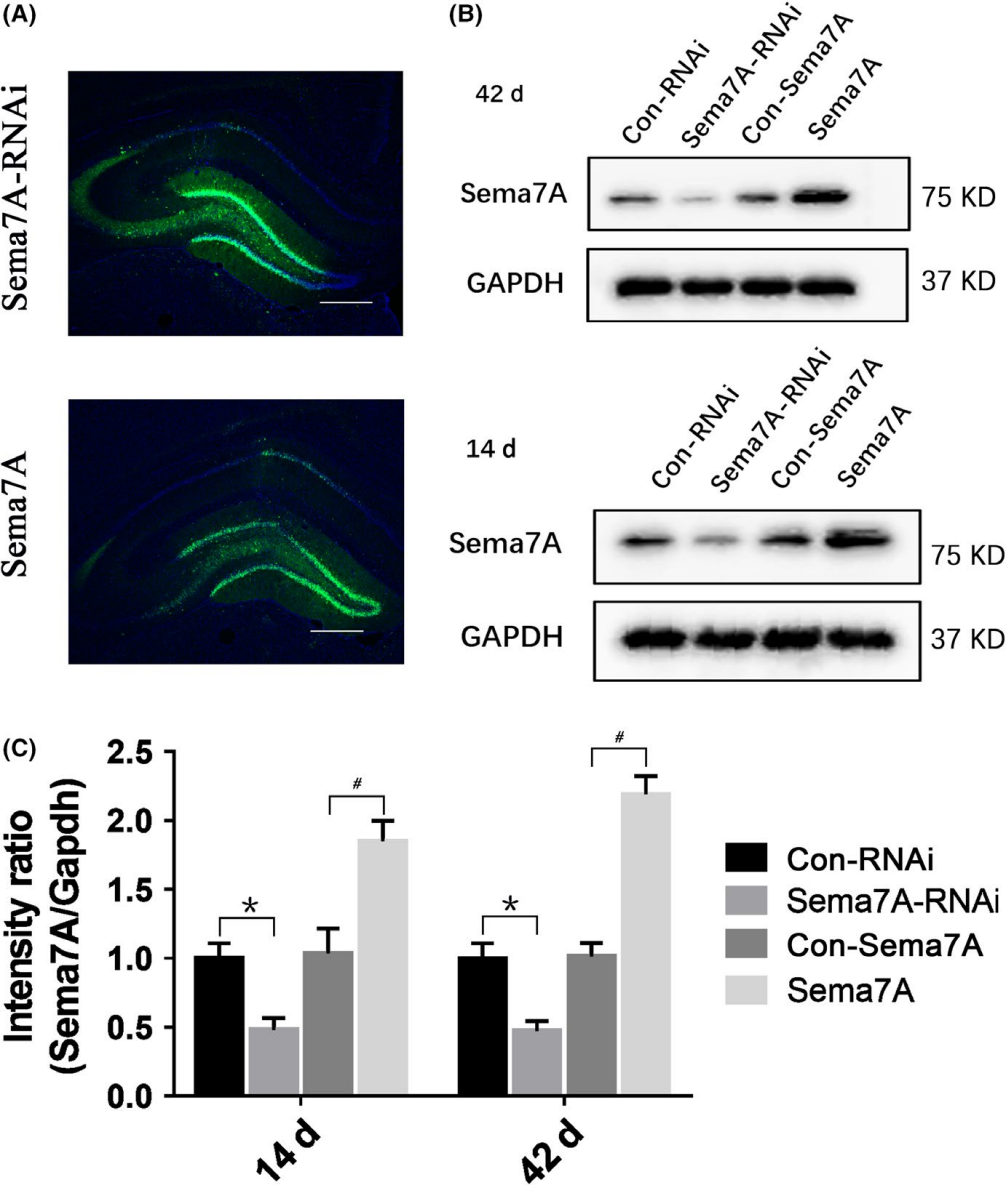
Expression of Sema7A and distribution of EGFP in the hippocampus after injection of LV vectors. A, Immunofluorescence image of EGFP expression in the dentate gyrus of the hippocampus at 14 d after stereotactic injection of lentivirus. DAPI staining (blue) represents the cellular nucleus (scale bar = 500 μm). B, Western blot images of Sema7A expression in the hippocampus at 14 and 42 days after injection of Sema7A and Sema7A‐RNAi. C, At different time points after injection, the mean intensity of Sema7A was decreased significantly in the Sema7A‐RNAi group and increased significantly in the Sema7A group. (*P < 0.05, compared with Con‐RNAi group; ^#^ *P* < 0.05, compared with Con‐Sema7A group, one‐way ANOVA test, n = 5)

The function and mechanisms of Sema7A in epilepsy were investigated using a lentivirus delivery system to regulate the level of Sema7A protein expression. The recombinant lentivirus vectors Sema7A and Sema7A‐RNAi were stereotaxically injected into the bilateral hippocampus of rats. To verify the efficiency and stability of Sema7A expression mediated by lentivirus, we observed the distribution of EGFP and measured the expression of Sema7A in the hippocampus after lentivirus injection. EGFP‐positive cells were mainly neurons localized in the DG (Figure 5A), and the protein expression of Sema7A was significantly decreased at 14 and 42 days after Sema7A‐RNAi injection compared with that in the Con‐RNAi group. Furthermore, the protein expression of Sema7A was significantly increased at 14 and 42 days after Sema7A injection (Figure 5B, 5), indicating that the two lentiviruses, Sema7A and Sema7A‐RNAi, had been successfully transfected into the hippocampal neurons and effectively altered the protein level of Sema7A in the rat hippocampus.

### 3.5 Sema7A affects seizure susceptibility and activity

**Figure 6:**
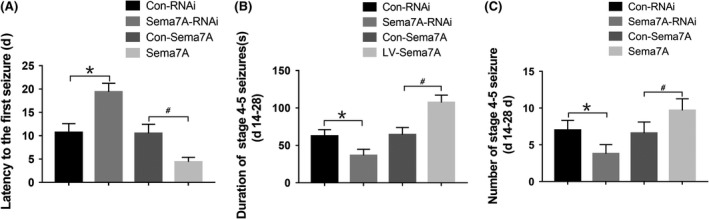
Effect of Sema7A on seizure susceptibility and activity in PTZ‐kindled rats. A, The mean latency to the first seizure was increased significantly in the Sema7A‐RNAi group and decreased in the Sema7A group. B, C, The mean duration (B) and number of seizures (C) were decreased in the Sema7A‐RNAi group and increased in the Sema7A group. (*P < 0.05, compared with Con‐RNAi group; ^#^ *P* < 0.05, compared with Con‐Sema7A group, one‐way ANOVA test, n = 10)

Fourteen days after the intracranial injection of lentivirus, a subconvulsant dose (35 mg/kg) of PTZ was intraperitoneally injected daily for behavioral evaluation. A significant increase in latency was observed (*P* < 0.05; Figure 6A), while both the total duration and the number of seizures were decreased in the Sema7A‐RNAi group compared with that in the Con‐RNAi group (*P* < 0.05; Figure 6B, 6). In addition, we also investigated whether overexpression of Sema7A could have an adverse effect on rat behavior. A significantly reduced latency was observed in the Sema7A group compared with that in the Con‐Sema7A group (*P* < 0.05; Figure 6A), while the duration and number of seizures were increased (*P* < 0.05; Figure 6B, 6). All results suggested that knocking down Sema7A in the rat hippocampus could reduce seizure susceptibility, while overexpression of Sema7A could increase the susceptibility to seizures and promote epileptogenesis.

### 3.6 Effect of Sema7A on mossy fiber projection of the hippocampus

**Figure 7:**
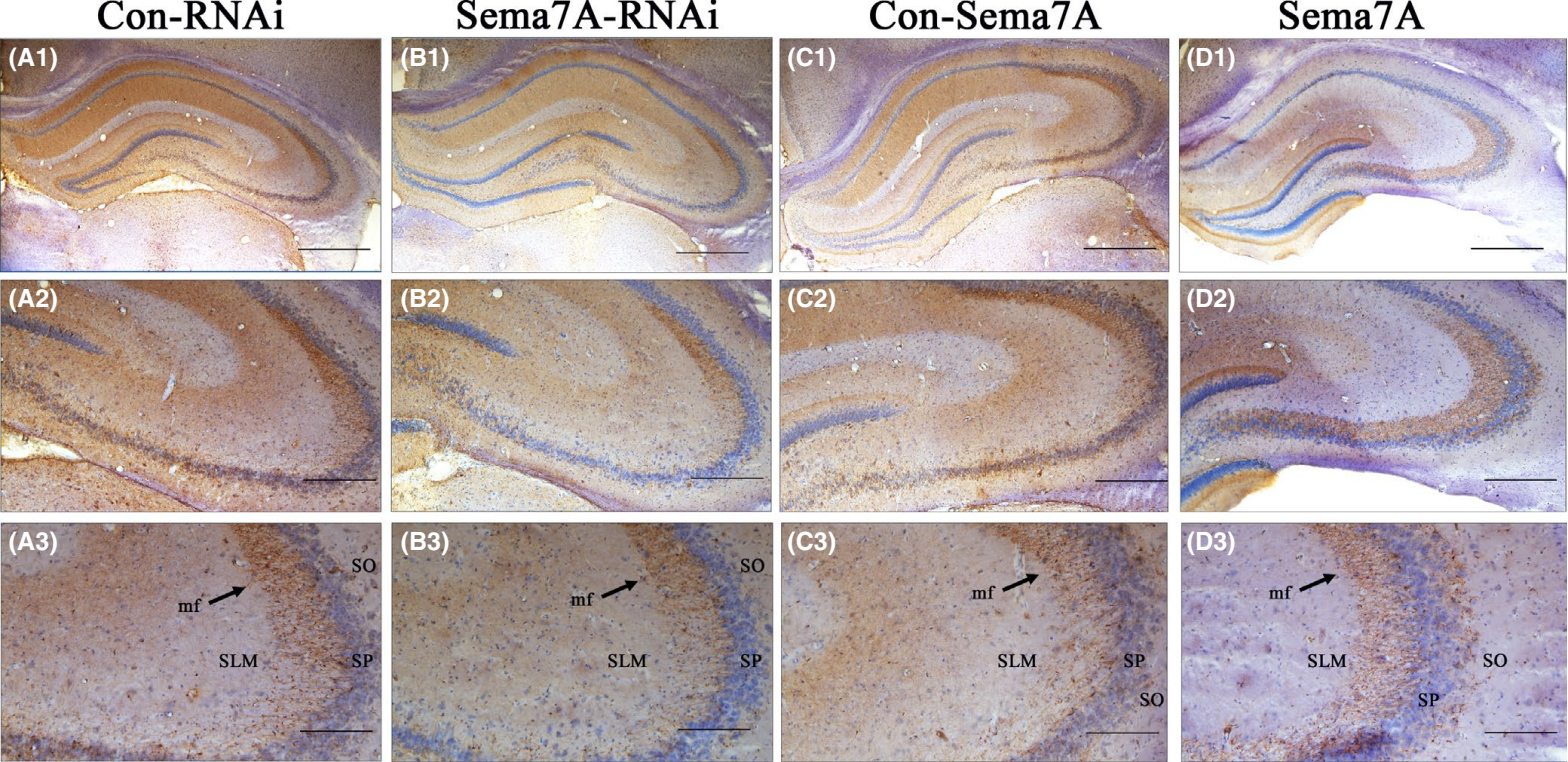
Mossy fiber (mf) projections in the DG of different groups after PTZ kindling. Mossy fiber projections to the CA3 field reveal significant defects in the Sema7A‐RNAi group (B) compared with that in the Con‐RNAi group (A). Compared with the Con‐Sema7A group (C), the Sema7A group (D) shows increased numbers of mossy fibers projecting from the hilus to CA3, deeply penetrating into the stratum pyramidale (SP) of CA3, and in some instances, extending into the stratum oriens (SO). The labels 1, 2, and 3 represent different magnifications. The scale bar is 1.0 mm in (A1‐D1), 200 µm in (A2‐D2), and 100 µm in (A3‐D3)

Previous studies suggest that Sema7A is an axonal guidance regulator.^29^ In this study, we investigated the effect of Sema7A on the growth of hippocampal mossy fibers in epileptic models. After 2 weeks of stereotaxic injection of BDA into the hippocampal DG region of rats, BDA‐positive fibers were detected to assess the development of mossy fiber projections from point to point. We examined the mossy fiber projections from the DG to CA3 in rats. In the control group, the main axons of dentate granule cells (mossy fibers) grew along the inner surface of CA3 in the stratum lacunosum‐moleculare (SLM) layer, while only a few grew into the distal layer (Figure 7A, 7). However, in the Sema7A group, substantially more mossy fibers projected from the hilus to the CA3 SLM, and grew abnormally far laterally in the CA3 field, deeply penetrating the pyramidal (SP) layer of CA3 and, in some cases, extending to the stratum oriens (SO; Figure 7D).The mossy fiber staining in the CA3 region was much stronger in the Sema7A group than in the Con‐Sema7A group, while that in the CA3 region was fainter in the Sema7A‐RNAi group (Figure 7B) than in the Con‐RNAi group. These results suggest that downregulation of Sema7A in the DG region inhibits mossy fiber projections, while upregulation of Sema7A promotes the projections.

### 3.7 Sema7A induced changes in ERK phosphorylation and inflammatory cytokines

**Figure 8:**
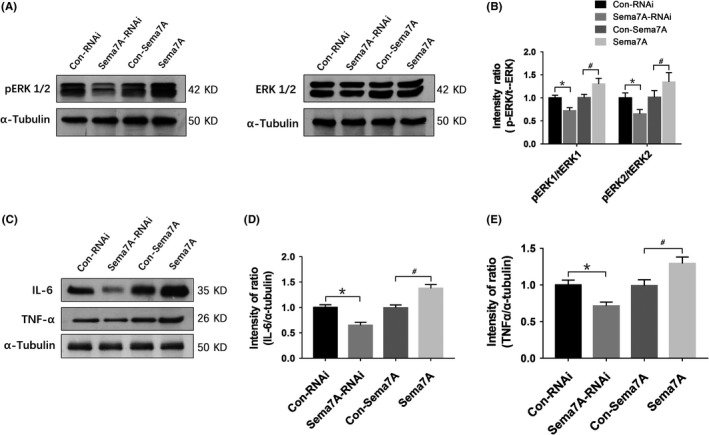
Sema7A affects pERK1/2 levels and the expression of IL‐6 and TNF‐α in the hippocampus. A, B, Western bands and bars of pERK1/2 and ERK1/2 in Sema7A‐RNAi and Sema7A rats after PTZ kindling. The mean intensity ratios of pERK1/2 significantly were decreased in the Sema7A‐RNAi group and increased in the Sema7A group. C, D, E, Western bands and bars for IL‐6 or TNF‐α in the Sema7A‐RNAi and Sema7A groups after PTZ kindling. The mean intensity ratios of IL‐6 (D) showed significantly decreased expression in the Sema7A‐RNAi group and increased expression in the Sema7A group. The same trend was observed in TNF‐α expression (E). (*P < 0.05, compared with Con‐RNAi group; ^#^ *P* < 0.05, compared with Con‐Sema7A group, one‐way ANOVA test, n = 5)

In PTZ‐kindled epileptic rats, we assessed the phosphorylation of ERK 1/2 and the levels of the inflammatory cytokines IL‐6 and TNF‐α after Sema7A knockdown or overexpression. The expression levels of pERK1/tERK1, pERK2/tERK2 (Figure 8A, 8), IL‐6, and TNF‐α (Figure 8C‐E) were significantly decreased in the Sema7A‐RNAi group compared with those in the Con‐RNAi group (*P* < 0.05), while their levels were significantly increased in the Sema7A group compared with those in the Con‐Sema7A group (*P* < 0.05).

### 3.8 U0126 suppresses seizure severity and reduces the inflammatory response in Sema7A‐overexpressing rats

**Figure 9:**
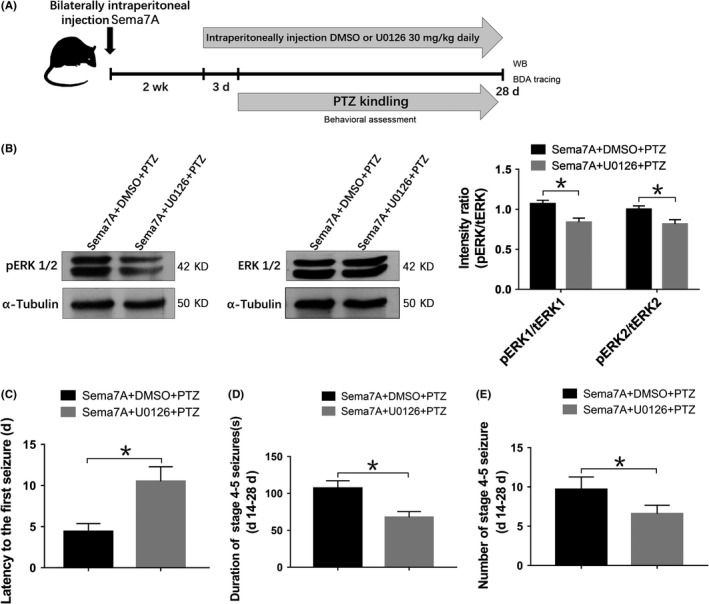
U0126 suppresses pERK1/2 expression and seizure severity after Sema7A overexpression in epileptic rats. A, Schematic diagram showing the design of the U0126 experiment in Sema7A‐transduced rats. B, Western bands and bars of pERK1/2 and ERK1/2 in Sema7A + DMSO + PTZ and Sema7A + U0126+PTZ groups. The intensity ratios of pERK1/2 were significantly decreased in the Sema7A + U0126 + PTZ group (*P < 0.05, compared with Sema7A + DMSO + PTZ group, unpaired *t* test, n = 5). C‐E, Behavioral tests showed increased latency to the first seizure (C), reduced duration, D, and reduced number of seizures (E) in the Sema7A + U0126 + PTZ group (*P < 0.05, compared with Sema7A + DMSO + PTZ group, unpaired *t* test, n = 10)

To determine whether ERK activation is involved in the function of Sema7A in epilepsy, we detected the levels of IL‐6 and TNF‐α in the presence of U0126 after injecting the lentiviral vector Sema7A (Figure 9A), and the phosphorylation levels of ERK1 and ERK2 were significantly inhibited in the U0126‐treated group compared with those in the DMSO‐treated group after PTZ kindling (*P* < 0.05; Figure 9B). Analysis of the behavioral performance showed that the latency was significantly increased (*P* < 0.05; Figure 9C) and that the duration and number of seizures were decreased in the U0126‐treated group in comparison with those in the DMSO‐treated group. (*P* < 0.05; Figure 9D, 9). These results indicate that U0126 could alleviate seizure susceptibility and severity by effectively inhibiting ERK1/2 phosphorylation.

**Figure 10:**
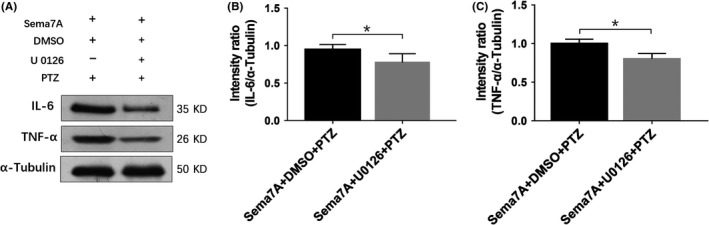
U0126 suppresses cytokines IL‐6 and TNF‐α after Sema7A overexpression in epileptic rats. Western images A, and bars B, C, of IL‐6 and TNF‐α in the Sema7A + DMSO + PTZ and Sema7A + U0126 + PTZ groups. The mean intensity ratios showed significantly decreased expression of IL‐6 (B) and TNF‐α (C) in the Sema7A + U0126 + PTZ group. (*P < 0.05, compared with the DMSO‐treated group, unpaired *t* test, n = 5)

We also examined the changes in the inflammatory cytokines IL‐6 and TNF‐α after U0126. In the Sema7A + U0126 + PTZ group, the protein levels of IL‐6 and TNF‐α were significantly decreased compared with those in the Sema7A + DMSO + PTZ group (Figure 10A, 10; *P* < 0.05), which indicated that the inhibitor U0126 could reduce the production of IL‐6 and TNF‐α in PTZ‐kindled rats. These results further suggest that Sema7A could affect the production of inflammatory cytokines by regulating ERK1/2 phosphorylation in epileptic rat models.

**Figure 11:**
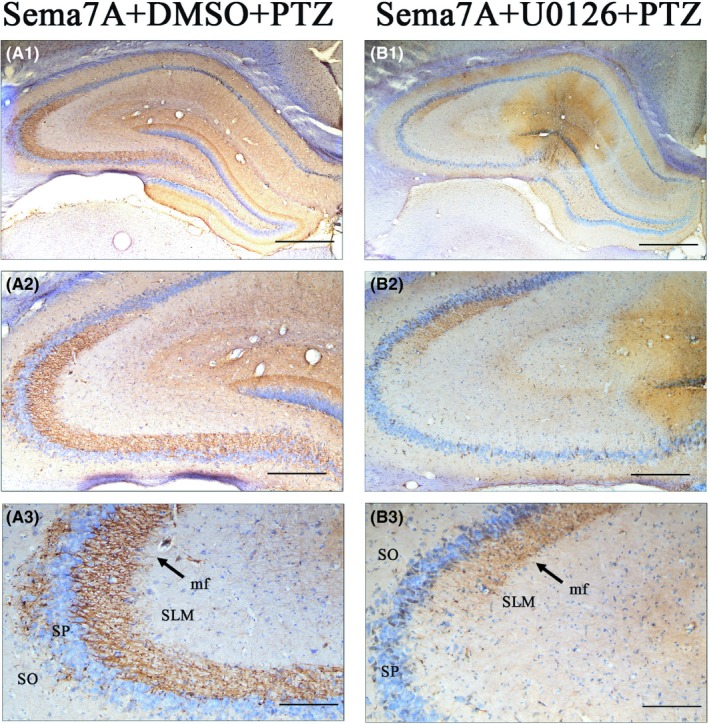
U0126 inhibits mossy fiber (mf) projections. Rats in Sema7A + DMSO + PTZ group exhibited numerous mossy fibers projecting to the CA3 field, extending deeply into the SP and growing beyond into the SO (A). Furthermore, compared with those in the Sema7A + DMSO + PTZ group, rats in Sema7A + U0126 + PTZ group exhibited a reduced number of mossy fibers, which projected into the CA3 but did not extend deeply (B). The labels 1, 2, and 3 represent different magnifications. The scale bar is 1.0 mm in (A1‐B1), 200 µm in (A2‐B2), and 100 µm in (A3‐B3)

Previous studies have shown that Sema7A not only relates to the inflammatory immune response but also regulates axon growth via the MAPK signaling pathway. Therefore, we investigated effects of U0126 on mossy fibers in rats overexpressing Sema7A. In Sema7A + U0126 + PTZ group, the distribution of mossy fibers in the hippocampus was significantly reduced compared with Sema7A + DMSO + PTZ group (Figure 11). These results indicated that Sema7A could act via the ERK signaling pathway to promote the aberrant development of hippocampal mossy fiber projections in an epileptic rat model.

## 4 DISCUSSION

The expression of Sema7A in the CNS increases as development progresses.^27^ As we report, Sema7A localizes to the cytomembrane and cytoplasm of neuronal subsets in the hippocampus and temporal neocortex. Interestingly, a significant increase in the expression of Sema7A was found in the temporal neocortex of patients with intractable epilepsy and PTZ‐kindled epileptic rats. Given the abnormal expression of Sema7A, we hypothesized that Sema7A might play an important role in TLE, and behavioral analysis confirmed this role. Overexpression of Sema7A in the hippocampus increases seizure susceptibility and severity in PTZ‐treated rats, whereas knockdown of Sema7A results in decreased seizure susceptibility and severity. Therefore, our data indicate that Sema7A contributes to epileptic seizures. As this study is the first on the role of Sema7A in epilepsy, further investigations are needed to elucidate the possible mechanistic role of this protein in epilepsy.

Sema7A is considered an important effector molecule in T cell‐mediated inflammation. By stimulating the production of cytokines in monocytes and macrophages by binding α1β1 integrin, Sema7A plays a critical role in the effector stage of the inflammatory immune response.^26^ Sema7A is a potent monocyte activator that can stimulate the production of chemotaxis and inflammatory cytokines, such as TNF‐alpha and IL‐6.^25^ In the pathogenesis of multiple sclerosis (MS), Sema7A participates in peripheral immunity and CNS inflammation and promotes cytokines, such as IL‐6 and TNF‐α.^28^ Increasing evidence highlights that inflammation caused by brain damage may be involved in epileptogenesis. Because brain inflammation in epilepsy is not merely a pathological phenomenon but may also be related to the mechanisms of neuronal hyperexcitability, it can potentially be considered a biomarker of disease development and severity.^43^ Several studies have revealed increased levels of inflammatory mediators (eg, cytokines such as IL‐6 and TNF‐α) in epilepsy.^44^ TNF‐α and IL‐6 may increase the excitability of neural circuits and reduce the seizure threshold by triggering a cascade of reactions.^45^ TNF‐α can activate the recruitment of AMPA receptors on neuronal membranes and induce the endocytosis of GABA_A_ receptors, thus increasing the average frequency of AMPA‐dependent miniature excitatory postsynaptic currents in hippocampal neurons and reducing the intensity of GABA_A_ inhibitory synapses.^46^, ^47^, ^48^ In our research, overexpression of Sema7A promoted further increases in TNF‐α and IL‐6 expression in PTZ‐kindled rats, but these increases were ameliorated by Sema7A knockdown, indicating that Sema7A may mediate seizure activity by increasing neuroinflammation in the brain during epilepsy.

As we described earlier, Sema7A exerts an important effect via the MAPKS pathway. In neuronal cells, Sema7A leads to increased expression of the inflammatory cytokines IL‐6 and TNF‐α by activating ERK1/2 phosphorylation, while the production of cytokines induced by Sema7A is inhibited by the MEK1/2 inhibitor U0126.^26^ The MAPK pathway is a crucial signaling pathway that transduces seizure activity,^49^ and ERK inactivation^50^, ^51^ and activation^52^ result in decreased and enhanced seizure activity, respectively. In this study, overexpression of Sema7A increased ERK1/2 phosphorylation, whereas knockdown of Sema7A significantly reduced ERK1/2 phosphorylation. Notably, inhibition of ERK phosphorylation by U0126 significantly decreased the susceptibility of rats to seizures and decreased the severity of seizures induced by overexpression of Sema7A. Furthermore, U0126 significantly reduced the production of the inflammatory factors IL‐6 and TNF‐α. These results suggest the important role of the Sema7A‐ERK pathway in regulating seizure activity during epilepsy. Epileptic seizures and inflammatory mediators can promote each other, forming a positive feedback loop.^53^

Many studies have shown that Sema7A is not only an immunoregulatory molecule but also a classical axon guidance cue that can promote axon growth.^27^, ^54^, ^55^ Sema7A promotes axon outgrowth by activating integrin receptors and MAPK signaling pathways.^29^ Mossy fibers are the most studied axons in epilepsy.^56^, ^57^, ^58^ In the hippocampus, mossy fibers converge in the hilus, branch out and connect with dendrites of hilar neurons, including mossy cells and pyramidal basket cells, and then project to the CA3 region.^59^ Aberrant synapses are formed by mossy fibers and dendrites of abnormally growing granule cells, leading to recurrent excitatory circuitry.^60^ In this study, we qualitatively observed the effect of Sema7A on mossy fiber projections in the hippocampus of an epileptic rat model. BDA tracing experimental results showed that the mossy fiber projections were increased in epileptic rats of the Sema7A overexpression group but decreased in the knockdown group, indicating that Sema7A can promote mossy fiber growth in epilepsy. After inhibition of ERK phosphorylation by U0126 in the Sema7A overexpression group, mossy fiber projections were significantly reduced in epileptic rats. These results are consistent with previous studies showing that Sema7A promotes the growth of brain axons via the ERK pathway, leading to abnormal neural circuits.

Knocking out Sema7A causes miniature excitatory postsynaptic current (mEPSCs), suggesting that Sema7A promotes excitatory inputs.^54^ In CNS, Sema7A promotes the growth of neurites and neuroinflammation by binding to β1‐Integrin receptor, which activated Ca2^+^ currents through effects on voltage‐sensitive calcium channels and excitatory NMDA currents in neurons.^61^, ^62^ These changes could enhance the glutamate excitability and burst activities in hippocampus, cause hyperexcitability in neurons, and could further lead to the development of the epilepsy.

This study is the first to explore the relationship between Sema7A and epilepsy and to further explore the inflammatory and morphological mechanisms of Sema7A‐mediated seizures in epilepsy. However, more studies on the mechanistic relationship between Sema7A and excitatory or inhibitory receptors are also needed.

In conclusion, our study demonstrates that Sema7A is upregulated in the brain tissues of refractory TLE patients and PTZ‐kindled model rats and plays an important role in epilepsy. In PTZ‐kindled rats, Sema7A may promote seizure activity, ERK phosphorylation, IL‐6 and TNF‐α production, and hippocampal mossy fiber projections. Sema7A may mediate seizure activity by regulating the ERK‐mediated inflammatory response and mossy fiber growth in epilepsy. Hence, we speculate that Sema7A may be a potential therapeutic target and provide possibilities for future clinical treatment.

## CONFLICTS OF INTEREST

The authors have no conflict of interest.

## ETHICAL APPROVAL

This research was approved by the ethics committees of Chongqing Medical University. The study procedures were performed in compliance with the Declaration of Helsinki. Informed consent for research purposes was obtained from all patients or their guardians.
